# Effectiveness of COVID-19 Vaccines in Adults with Diabetes Mellitus: A Systematic Review

**DOI:** 10.3390/vaccines11010024

**Published:** 2022-12-22

**Authors:** Jesse M. van den Berg, Sharon Remmelzwaal, Marieke T. Blom, Beryl A. C. E. van Hoek, Karin M. A. Swart, Jetty A. Overbeek, George L. Burchell, Ron M. C. Herings, Petra J. M. Elders

**Affiliations:** 1 Department of General Practice, Amsterdam UMC Location Vrije Universiteit Amsterdam, De Boelelaan 1117, 1081 HV Amsterdam, The Netherlands; 2 Amsterdam Public Health, Health Behaviors & Chronic Diseases, Amsterdam, The Netherlands; 3 PHARMO Institute for Drug Outcomes Research, 3528 AE Utrecht, The Netherlands; 4 Department of Epidemiology and Data Science, Amsterdam UMC Location Vrije Universiteit Amsterdam, De Boelelaan 1117, 1081 HV Amsterdam, The Netherlands; 5 Medical Library, Vrije Universiteit Amsterdam, De Boelelaan 1117, 1081 HV Amsterdam, The Netherlands

**Keywords:** COVID-19, SARS-CoV-2, coronavirus, vaccine effectiveness, diabetes, vaccination, systematic review

## Abstract

Persons with diabetes mellitus may have an increased risk of severe illness or death from COVID-19 compared to persons without diabetes. Prior studies indicate that immune response and thus vaccine effectiveness might be lower in persons with diabetes. We aimed to systematically review the effectiveness of COVID-19 vaccines in adults with diabetes. Pubmed, Embase, Web of Science and Cochrane Library were searched for studies that evaluated the effectiveness of COVID-19 vaccines in adults with diabetes, published before 4 March 2022. Risk of bias in the included studies was evaluated using the ROBINS-I tool. At least two reviewers conducted the study selection, data extraction, and risk of bias assessment independently. After screening of 2196 studies, a total of 17 articles were included. Six different COVID-19 vaccines (Ad5-nCoV-S, AZD1222, BNT162b2, CoronaVac, JNJ-78436735, and mRNA-1273) were included in the synthesis. Vaccine effectiveness was reported for SARS-CoV-2 infection, symptomatic COVID-19, hospitalization, and death, and ranged from 24 to 96% in persons with diabetes, and from 33 to 97% in total study populations; effectiveness was generally lower for persons with diabetes. Odds ratios for breakthrough infection or severe COVID-19 ranged from 1.03 to 2.41 in vaccinated persons with diabetes compared to persons without diabetes. Even though the included studies were very heterogeneous, results from the synthesis indicate that effectiveness of COVID-19 vaccines might be lower in persons with diabetes. More research is needed on the comparison of vaccine effectiveness between persons with and without diabetes, and the effectiveness of repeat COVID-19 vaccinations.

## 1. Introduction

Several studies have shown an increased risk of severe illness or mortality from COVID-19 for persons with diabetes, compared to persons without diabetes [1,2,3]. Therefore, vaccination against SARS-CoV-2 is most needed to prevent spreading and to lower the chance of severe illness. In the past years, several different vaccines have been developed that are all efficacious in preventing both infection with SARS-CoV-2 and severe COVID-19 complications in adults [4,5,6,7]. Although people with diabetes were included in at least some of these trials, the efficacy was not investigated separately for people with diabetes. The need for data on effectiveness of COVID-19 vaccination in people with diabetes has been stressed in several studies [8,9]. It could have implications on policy regarding prioritizing and considering additional vaccinations in this population.

People with diabetes have an increased risk of infections [10,11]. This is related to chronic inflammation caused by high blood glucose levels and pro-inflammatory mediators. This chronic inflammation, which is also obesity related, may cause dysfunction of immune response and could explain increased incidence of infection with SARS-CoV-2 in people with diabetes [12]. This impaired immune response may also reduce the effectiveness of COVID-19 vaccination. It has already been shown that effectiveness of COVID-19 vaccination can be lower in older or immunocompromised patients [13,14]. Prior studies indicate that vaccination (e.g., against seasonal influenza or hepatitis B) might be less effective in people with diabetes [15,16]. A recent systematic review showed an impaired antibody response following COVID-19 vaccination in people with diabetes compared to healthy controls [17]. These findings suggest that effectiveness of COVID-19 vaccination may also be lower in people with diabetes. To our knowledge, the effectiveness of COVID-19 vaccinations in people with diabetes has not yet been systematically reviewed.

In this study, we aimed to perform a systematic review investigating the effectiveness of COVID-19 vaccination among adults with diabetes in terms of preventing SARS-CoV-2 infection, symptomatic COVID-19, hospitalization, and mortality.

## 2. Materials and Methods

### 2.1. Search Strategy

This systematic review was performed in compliance with the Preferred Reporting Items for Systematic Reviews and Meta-analyses (PRISMA) guideline. The study protocol was registered with the Prospective Register of Systematic Reviews (PROSPERO; CRD42022315360).

A systematic search was performed in PubMed, Embase, Clarivate Analytics/Web of Science Core Collection and the Wiley/Cochrane Library. The search period within the databases was from inception to 4 March 2022 and conducted by G.L.B. and J.M.v.d.B. using included keywords and free text terms for (synonyms of) ‘COVID-19 vaccination’ combined with (synonyms of) ‘diabetes’ or (synonyms of) ‘co-morbidity’. An overview of the search terms per database is presented in the Supplementary Materials (see Tables S1–S4). No limitations on date or language were applied in the search.

### 2.2. Eligibility Criteria

After removal of duplicates, the remaining publications were screened for eligibility using the following inclusion and exclusion criteria. We included studies on effectiveness of any COVID-19 vaccine. We included (1) studies comparing outcome rates in vaccinated persons with diabetes to unvaccinated persons with diabetes and (2) studies comparing breakthrough infection or severe COVID-19 in vaccinated persons with diabetes compared to vaccinated persons without diabetes. The following studies were excluded: (1) animal experiments, case reports, letters and editorials, reviews; (2) no people with diabetes; (3) no information on outcomes in people with diabetes; (4) other outcomes; and (5) other comparisons as defined in the study protocol.

### 2.3. Study Selection

Four independent reviewers (J.M.v.d.B., and M.T.B., B.A.C.E.v.H. or S.R.) performed screening and selection of the studies by screening title and abstract and subsequently, the full texts of the remaining studies. All potentially eligible studies were reviewed by at least two reviewers. Disagreement was resolved through discussion with all four reviewers to reach consensus.

### 2.4. Data Collection

Two of three independent investigators (J.M.v.d.B., and either M.T.B. or S.R.) extracted data from the included studies. The following data were collected, if available: (1) study characteristics: first author, publication year, country, study design, and reported outcomes; (2) patient characteristics: sample size, age, sex, information on diabetes (number of patients, type, duration, source of diagnosis), and comorbidities; (3) vaccine characteristics: type, dose, and number of vaccinated patients; (4) outcomes of studies on vaccine effectiveness (VE): number of patients with and without SARS-CoV-2 infection, symptomatic COVID-19, hospitalization, deaths, unadjusted and adjusted risk ratios (RR) for these outcomes, unadjusted and adjusted vaccine effectiveness for these outcomes; and (5) outcomes of studies on breakthrough infection or severe COVID-19 after vaccination: number of patients with and without breakthrough infection or severe COVID-19, and unadjusted and adjusted odds ratios for these outcomes.

### 2.5. Outcome Measures

Outcomes included VE, in terms of effectiveness of vaccination preventing SARS-CoV-2 infection, symptomatic COVID-19, hospitalization, and mortality. VE was defined as (1-RR)*100%, where RR is the rate of the outcome in vaccinated patients divided by the rate of the outcome in unvaccinated patients. We reported the VE estimates with 95% confidence intervals for multiple vaccines separately within studies, if possible. We reported VE for both people with diabetes and for either total study population or people without diabetes, whichever was available per study.

As an indirect outcome of VE we reported the odds ratio (OR) for breakthrough infection or severe COVID-19 in vaccinated persons with diabetes, compared to vaccinated persons without diabetes.

### 2.6. Quality Assessment

Two of three independent reviewers (J.M.v.d.B., and either M.T.B. or S.R.) assessed the risk of bias of the included studies using the ROBINS-I tool (Risk of Bias In Non-randomized Studies of Interventions) [18], which evaluates risk of bias considering the following domains: (1) confounding; (2) selection of participants; (3) classification of interventions; (4) deviations from intended interventions; (5) missing data; (6) measurement of outcomes; and (7) selection of reported result. For each of these domains, risk of bias was scored as low, moderate, serious, critical, or no information. Considering the scores for all domains, overall risk of bias was scored in the same gradation.

### 2.7. Synthesis Methods

Extracted study characteristics were summarized in tabular form and results of included studies were summarized in forest plots. We would perform meta-analyses for VE regarding all outcomes in both people with diabetes and in total study population if we could consider at least three studies to have comparable outcomes, and the studies were sufficiently homogeneous in terms of study design, study population, vaccine type, vaccine dose, and time period. If these criteria were met, we would assess statistical heterogeneity.

## 3. Results

### 3.1. Literature Search

**Figure 1:**
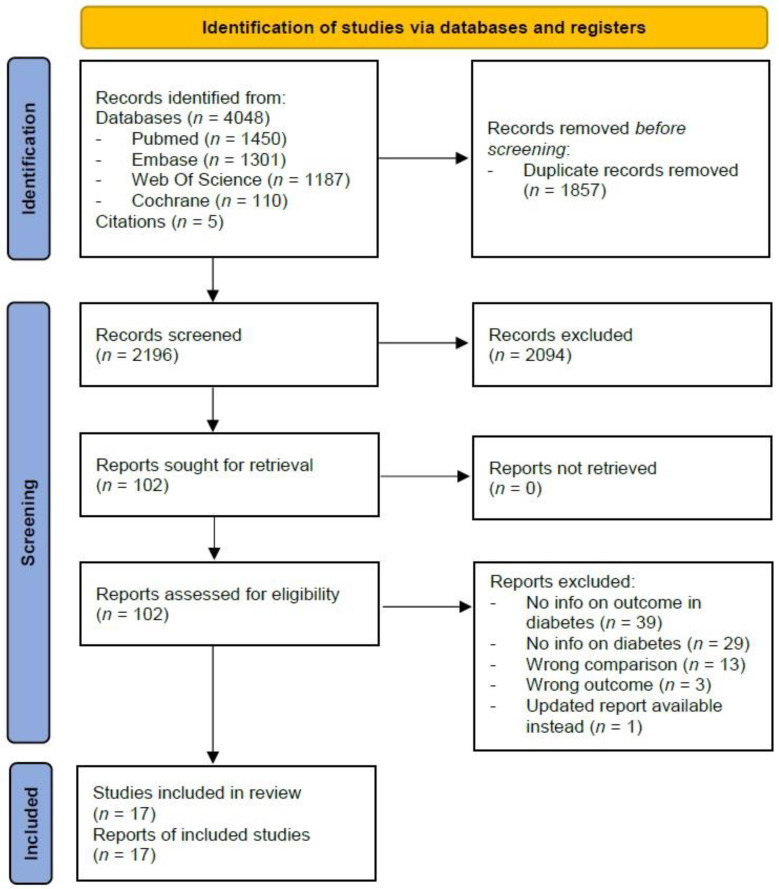
Flowchart of inclusion of studies.

In total, we identified 4048 studies, of which 1450 from PubMed, 1301 from Embase, 1187 from Web Of Science, and 110 from Cochrane Library. After excluding 1857 duplicates and screening of title and abstract of the remaining 2191 studies, we retained 97 full texts that met our inclusion criteria for review of eligibility. After exclusion of irrelevant studies, due to various reasons such as missing outcome of interest or missing information on people with diabetes, 12 studies [19,20,21,22,23,24,25,26,27,28,29,30] were included in the review. Through citation searching of included articles, five additional studies [31,32,33,34,35] were identified and included, resulting in a total of 17 studies (Figure 1).

### 3.2. Study Characteristics

A total of 16,587,405 patients were included from 17 studies, from nine countries. The abstracted data are summarized in Table 1. Six different COVID-19 vaccines were administered: Ad5-nCoV-S (CanSino), AZD1222 (AstraZeneca), BNT162b2 (Pfizer-BioNTech), CoronaVac (Sinovac), JNJ-78436735 (Janssen), and mRNA-1273 (Moderna). BNT162b2 was administered most frequently, in 15 of 17 studies. Vaccination protocol was not always completed: in 12 studies, a proportion of, or all participants received only one of two doses during the study period. The mean age of participants ranged from 37 to 76 years.

### 3.3. Risk of Bias

There was evidence of low risk of bias in two studies, moderate risk in three, serious risk in eight, critical risk in three, and no information on risk of bias in one study (Figure S1). Overall judgement indicating serious or critical risk of bias were mainly caused by increased risk of bias in domains concerning bias due to confounding and bias due to selection of patients. Concerning confounding bias, we considered only five out of 17 studies to have low risk of bias. Risk of bias was considered high if they did not appropriately control for potential confounders or did not present adjusted estimates at all. Concerning selection bias, we considered more than half of all studies to have a moderate to critical risk of bias. For almost all studies, there was little to no information on domains concerning bias due to missing data or bias due to deviations from intended interventions (Figure S2).

### 3.4. Outcomes

For three outcomes—preventing infection, symptomatic illness and hospitalization—there were at least three studies per outcome. However, we considered the studies to be too heterogeneous in terms of study design, study population, vaccine type, vaccine dose, and time period and therefore refrained from performing a meta-analysis. Instead, we performed a narrative descriptive synthesis.

**Figure 2:**
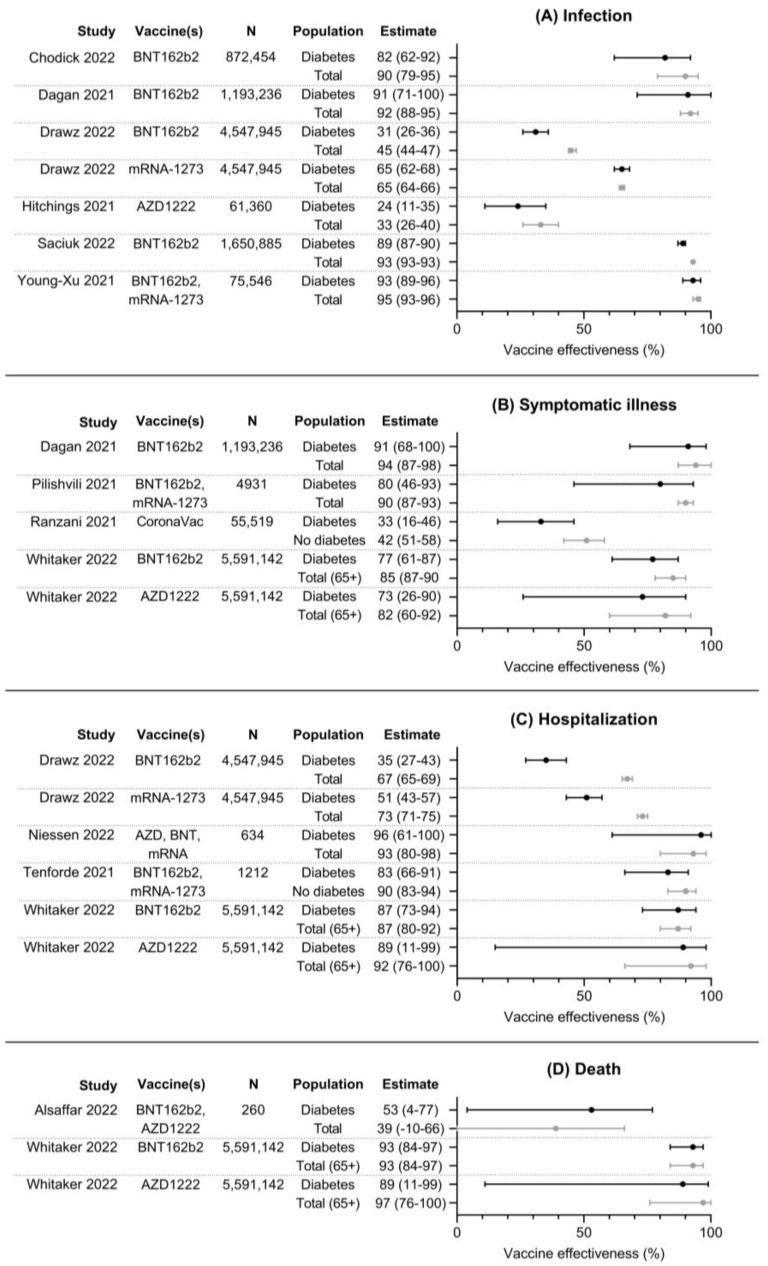
Forest plots with point estimates and 95% confidence intervals of effectiveness of COVID-19 vaccines against (**A**) infection, (**B**) symptomatic illness, (**C**) hospitalization, and (**D**) death. N denotes total number of participants per study, as numbers per group were usually not available [20,22,23,25,26,27,29,31,32,33,34,35].

The results of 12 included studies investigating vaccine effectiveness (VE), comparing event rates in vaccinated patients to unvaccinated patients, are summarized in Figure 2. VE for SARS-CoV-2 infection was investigated in six studies, VE for symptomatic COVID-19 in four studies, VE for hospitalization in four studies, and VE for death in two studies. VE for infection with SARS-CoV-2 ranged from 24 to 93% in people with diabetes and from 33 to 95% in the total population. VE estimates were consistently lower in persons with diabetes compared to the total population, but confidence intervals overlapped in most studies. VE for symptomatic COVID-19 ranged from 33 to 91% in persons with diabetes and from 42 to 94% in the total population. Similarly, VE estimates were lower for persons with diabetes in all three studies compared to the total population or persons without diabetes. VE for hospitalization ranged from 35 to 96% in persons with diabetes and from 67 to 93% in the total population or persons without diabetes. In three of four studies, VE estimates were lower in persons with diabetes compared to the total population, with large and significant differences in one study [22]. VE for death ranged from 53 to 93% in persons with diabetes and from 39 to 97% in the total population. No clear differences in VE can be found between these groups.

**Figure 3:**
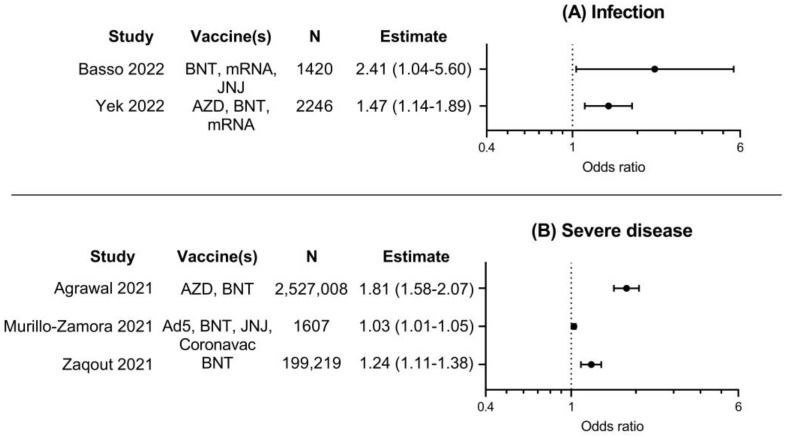
Forest plots with point estimates and 95% confidence intervals of odds ratios for A. infection with COVID-19, or B. severe COVID-19 for vaccinated persons with diabetes, compared to vaccinated persons without diabetes. N denotes total number of participants per study, as numbers per group were usually not available. Ad5, Ad5-nCoV-S (CanSino); AZD, AZD-1222 (AstraZeneca); BNT, BNT162b2 (Pfizer); JNJ, JNJ-78436735 (Janssen); mRNA, mRNA-1273 (Moderna) [19,21,24,28,30].

Results of five included studies investigating risk of breakthrough infection or severe illness in vaccinated persons, comparing persons with diabetes to persons without diabetes, are summarized in Figure 3. Risk for SARS-CoV-2 breakthrough infection was investigated in two studies, and risk for severe COVID-19 in three studies. The results of these studies were consistent; odds ratios for these outcomes ranged from 1.03 to 2.41 and were all significantly higher in vaccinated persons with diabetes. This indicates that COVID-19 vaccines might be less effective in preventing infection or severe illness for vaccinated persons with diabetes compared to vaccinated persons without diabetes.

## 4. Discussion

To our knowledge, this is the first systematic review to investigate the effectiveness of COVID-19 vaccines in persons with diabetes in terms of preventing SARS-CoV-2 infection, symptomatic COVID-19 infections, as well as complications resulting in hospitalization and death. A total of 17 studies were included, of which 12 studies investigated the outcomes in vaccinated versus unvaccinated people with diabetes, whilst five studies evaluated the risk of either SARS-CoV-2 breakthrough infection or COVID-19 complications among vaccinated persons with diabetes versus vaccinated persons without diabetes.

The included studies were very heterogeneous and differed in outcome, study design and population, vaccine type and dose as well as time period. Therefore, meta-analyses could not be performed. Data from the included studies, however, indicate that vaccine effectiveness (VE) might be lower in persons with diabetes compared to either total study population or persons without diabetes since estimates of VE were consistently lower in persons with diabetes. This was especially so for VE for hospitalization and to a lesser extent in preventing infection and symptomatic illness. Even though VE estimates were usually lower in persons with diabetes, confidence intervals often overlapped. Furthermore, no clear differences were found for VE for death. In most studies, there was no comparison between persons with or without diabetes; instead, VE was reported for the total number of participants and for persons with diabetes, but usually not for persons without diabetes separately. The possible lower VE in persons with diabetes might be explained by lowered antibody response following COVID-19 vaccination in persons with diabetes, as Boroumand, et al., have shown in a recent systematic review [17].

Some studies investigated VE not by comparing outcomes in vaccinated versus unvaccinated patients, but rather by comparing the rate of either breakthrough infection or severe COVID-19 in vaccinated persons with diabetes to vaccinated persons without diabetes. While this is not a direct measure of VE, all five of these studies reported higher odds for these outcomes in vaccinated persons with diabetes compared to persons without diabetes [19,21,24,28,30]. These findings imply that COVID-19 vaccines may be less effective for persons with diabetes.

VE might be relatively lower in persons with diabetes but was still high in most studies. This indicates that vaccination is also effective in people with diabetes, though it needs to be investigated if additional vaccination may sufficiently increase protection against COVID-19. High VE was seen especially in included studies in which the vaccination protocol was completed, as is in line with results from clinical trials and effectiveness studies [4,5,6,7]. Lower VE than expected was reported in several studies, which can be attributable to multiple factors. First, for some studies, VE may have been evaluated during a time period in which the Omicron variant was dominant, which is known to lower VE [36,37]. Unfortunately, only a few studies specifically reported the dominant SARS-CoV-2 variant during the study period, so it was not possible to make a distinction of VE before and after the Omicron wave. Within studies however, the comparison between VE in persons with diabetes and study population is unlikely to be impacted by effects of time frames or different SARS-CoV-2 variants. Nonetheless, there may have been differences in exposure or timing of vaccination between persons with or without diabetes. Second, studies were performed in different countries, which would normally increase generalizability. However, during the course of the COVID-19 pandemic, multiple factors affecting VE varied greatly between countries, such as differences in health measures and the amount of infected people during the study period. Third, investigated populations differed greatly between studies and included health care workers, veterans, older persons, or total population. This might affect outcomes, e.g., a lower VE in a population of health care workers because of increased transmission risks due to higher exposure. All these factors need to be taken into consideration when comparing results between studies.

This systematic review has some limitations. First, only two of 17 studies were considered to have low risk of bias, which greatly impacts the quality of evidence of the findings. Second, as the studies were very heterogeneous, we did not perform meta-analyses, which might hamper the interpretability and generalizability of the findings. Third, there was almost no information on characteristics of persons with diabetes, including diabetes type and comorbidities such as obesity, renal disease and cardiovascular disease. This would give more insight into the risk factors for decreased VE within persons with diabetes, as these comorbidities are all common in persons with diabetes. However, included studies provide valuable insights into the effectiveness of COVID-19 vaccines in patients with diabetes regardless of these comorbidities. Also, we accept the heterogeneity in research groups, as it reflects the real-world data of persons with diabetes. Finally, more research needs to be performed on VE in persons with diabetes compared to persons without diabetes, and whether or not VE increases to levels comparable to total population after administration of booster vaccinations. Research has shown that COVID-19 booster vaccinations increase vaccine effectiveness [38]. However, it is still unclear whether this also applies to persons with diabetes.

## 5. Conclusions

This systematic review aimed to investigate the effectiveness of COVID-19 vaccines in adults with diabetes. Results from 17 studies indicate that vaccine effectiveness for infection, symptomatic illness and hospitalization might be lower in persons with diabetes, compared to either persons without diabetes or total study population. However, the included studies were heterogeneous, and the majority was considered to have increased risk of bias, which may limit the interpretation of these findings. More research with sufficient quality is needed to determine whether COVID-19 vaccines are indeed less effective in persons with diabetes, which might give directions for policy regarding the need for prioritizing and considering additional vaccinations in this population.

## Figures and Tables

**Table 1:** Characteristics of included studies.

Study	Country	Design	Persons (N)	Persons with DM (N, %)	Mean age, years ^e^	Women (%) ^e^	Vaccine Type(s)	Vaccine dose	COVID-19 Outcomes (in persons with DM)
Comparison: vaccinated patients with diabetes vs. unvaccinated patients without diabetes									
Alsaffar et al., 2022 [20]	Saudi Arabia	Retrospective record review	260	104 (40)	52	46	BNT162b2 AZD1222	1 or 2	Death
Chodick et al., 2022 [31]	Israel	Retrospective cohort study	872,454	104,152 (12)	52	52	BNT162b2	2	Infection Symptomatic illness
Dagan et al., 2021 [32]	Israel	Matched case-control study	1,193,236	131,541 (11)	45	50	BNT162b2	1 or 2	Infection Symptomatic illness
Drawz et al., 2022 [22]	US	Test-negative study, and cohort study	4,547,945	322,090 (7) ^c^	NR ^a^	NR	BNT162b2 mRNA-1273	2 ^d^	Infection Hospitalization
Hitchings et al., 2021 [23]	Brazil	Matched case-control study	61,360	10,627 (17)	67	58	AZD1222	1	Infection
Niessen et al., 2022 [25]	The Nether-lands	Test-negative case-control study	634	155 (24)	68	43	BNT162b2 AZD1222 mRNA-1273	1 or 2	Hospitalization
Pilishvili et al., 2021 [26]	US	Test-negative case-control study	4931	228 (5)	37	83	BNT162b2 mRNA-1273	1 or 2	Symptomatic illness
Ranzani et al., 2021 [33]	Brazil	Matched test-negative case-control study	55,519	11,847 (21)	76	56	CoronaVac	1 or 2	Symptomatic illness
Saciuk et al., 2022 [27]	Israel	Retrospective cohort study	1,650,885	136,777 (8)	NR ^a^	52	BNT162b2	2	Infection
Tenforde et al., 2021 [34]	US	Prospective observational case-control study	1212	388 (32)	NR ^a^	49	BNT162b2 mRNA-1273	1 or 2	Hospitalization
Whitaker et al., 2022 [35]	England, UK	Cohort study, and test negative case-control study	5,591,142	407,741 (7)	NR ^a^	NR	BNT162b2 AZD1222	1 or 2	Symptomatic illness Hospitalization Death
Young-Xu, 2021 [29]	US	Matched test-negative case-control study	75,546	25,429 (34)	NR ^a^	10	BNT162b2 mRNA-1273	1 or 2	Infection
Comparison: vaccinated patients with DM vs. vaccinated patients without DM									
Agrawal et al., 2021 [19]	Scotland, UK	Prospective cohort study	2,527,008	264,243 (10)	NR ^a^	55	BNT162b2 AZD1222	1	Severe disease
Basso et al., 2022 [21]	Italy	Retrospective cohort study	1420	38 (3) ^c^	47 ^c^	NR	BNT162b2 mRNA-1273 JNJ-78436735	2 ^b^	Breakthrough- infection
Murillo-Zamora et al., 2022 [24]	Mexico	Retrospective cohort study	1607	NR	50	57	JNJ-78436735 BNT162b2 Ad5-nCoV-S CoronaVac	1 or 2 ^b^	Pneumonia
Yek et al., 2022 [28]	US	Prospective cohort study	2246	633 (28)	NR ^a^	58	BNT162b2 AZD1222 mRNA-1273	2 ^d^	Severe disease
Zaqout et al., 2021 [30]	Qatar	Retrospective cohort study	199,219	39,994 (20)	42	42	BNT162b2	1 or 2	Infection

NR, not reported. DM, diabetes mellitus. ^a^ Mean age not reported for total number of participants. ^b^ Participants who received JNJ-78436735 vaccine received 1 dose. ^c^ As a proportion of vaccinated participants, not of all participants. ^d^ Some participants received booster dose. ^e^ As a proportion of all study participants.

## Data Availability

The data that support the findings of this study are available from the corresponding author, J.M.v.d.B. upon reasonable request.

## Supplementary Materials

The following supporting information can be downloaded at: https://www.mdpi.com/article/10.3390/vaccines11010024/s1, Table S1: Search strategy in PubMed; Table S2: Search strategy in Embase.com; Table S3: Search strategy in Clarivate Analytics/Web of Science Core Collection; Table S4: Search strategy in Wiley/Cochrane Library; Figure S1. Risk of bias by domain and overall, for each study separately; Figure S2. Risk of bias in included studies, by domain and overall.
