# Supplementary material for: Effectiveness of COVID-19 Vaccines in Adults with Diabetes Mellitus: A Systematic Review
Source: Vaccines (Basel). 2022 Dec 22;11(1):24. doi: 10.3390/vaccines11010024 (PMC9861646; doi:10.3390/vaccines11010024)
Supplement: Supplementary file 1 [file vaccines-11-00024-s001.zip › vaccines-2096989-supplementary.pdf]

Supplementary Materials

Table S1. Search strategy in PubMed.

Search	Query	Results
#4	#1 AND (#2 OR #3)	1,450
#3	"Comorbidity"[Mesh] OR Comorbidit*[tiab] OR "Co morbidit*"[tiab] OR "underlying dis*"[tiab] OR "co existing dis*"[tiab] OR "co occurring dis*"[tiab] OR "concurrent dis*"[tiab] OR "underlying condition*"[tiab] OR "co-existing condition*"[tiab] OR "co-occurring condition*"[tiab] OR "concurrent condition*"[tiab] OR Multimorbidit*[tiab] OR "Multi morbidit*"[tiab]	297,875
#2	"Diabetes Mellitus"[Mesh] OR "Hypoglycemic Agents"[Mesh] OR "Hypoglycemic Agents" [Pharmacological Action] OR Prediabete*[tw] OR diabetes[tw] OR NIDDM[tw] OR IDDM[tw] OR T1DM[tw] OR T2DM[tw] OR diabetic*[tw] OR insulin*[tw] OR metformin*[tw] OR rosiglitazon*[tw] OR troglitazon*[tw] OR pioglitazon*[tw] OR hypoglycem*[tw] OR hypoglycaem*[tw]	1,082,697
#1	"COVID-19 Vaccines"[Mesh] OR ((Covid*[tw] OR corona*[tw] OR ncov[tw] OR "SARS CoV"[tw] OR SARSCoV*[tw] OR Coronavirus[tw] OR Pfizer[tw] OR comirnaty[tw] OR spikevax[tw] OR moderna[tw] OR "mRNA 1273"[tw] OR "Ad26.COV*"[tw] OR Janssen[tw] OR Johnson[tw] OR 78436735*[tw] OR BNT162*[tw]) AND (vaccin*[tw]))	31,885

Table S2: Search strategy in Embase.com.

Search	Query	Results
#5	#4 NOT ('chapter'/it OR 'conference abstract'/it OR 'conference paper'/it OR 'conference review'/it OR 'editorial'/it OR 'erratum'/it OR 'letter'/it OR 'note'/it OR 'review'/it OR 'short survey'/it)	1,301
#4	#1 AND (#2 OR #3)	2,516
#3	'comorbidity'/exp OR (Comorbidit* OR ‘Co morbidit*’ OR ‘underlying dis*’ OR ‘co existing dis*’ OR ‘co occurring dis*’ OR ‘concurrent dis*’ OR ‘underlying condition*’ OR ‘co-existing condition*’ OR ‘co-occurring condition*’ OR ‘concurrent condition*’ OR Multimorbidit* OR ‘Multi morbidit*’):ti,ab,kw	522,745
#2	'diabetes mellitus'/exp OR 'antidiabetic agent'/exp OR (Prediabete* OR diabetes OR NIDDM OR IDDM OR T1DM OR T2DM OR diabetic* OR insulin* OR metformin* OR rosiglitazon* OR troglitazon* OR pioglitazon* OR hypoglycem* OR hypoglycaem*):ti,ab,kw	1,721,381
#1	'SARS-CoV-2 vaccine'/exp OR ((Covid* OR corona* OR ncov OR ‘SARS CoV’ OR SARSCoV* OR Coronavirus OR Pfizer OR comirnaty OR spikevax OR moderna OR ‘mRNA 1273’ OR ‘Ad26.COV*’ OR Janssen OR Johnson OR 78436735* OR BNT162*) AND (vaccin*)):ti,ab,kw	33,038

Table S3: Search strategy in Clarivate Analytics/Web of Science Core Collection.

Search	Query	Results
#4	#1 AND (#2 OR #3)	1,187
#3	TS=(Comorbidit* OR "Co morbidit*" OR "underlying dis*" OR "co existing dis*" OR "co occurring dis*" OR "concurrent dis*" OR "underlying condition*" OR "co-existing condition*" OR "co-occurring condition*" OR "concurrent condition*" OR Multimorbidit* OR "Multi morbidit*")	251,769
#2	TS=(Prediabete* OR diabetes OR NIDDM OR IDDM OR T1DM OR T2DM OR diabetic* OR insulin* OR metformin* OR rosiglitazon* OR troglitazon* OR pioglitazon* OR hypoglycem* OR hypoglycaem*)	1,216,530
#1	TS=((Covid* OR corona* OR ncov OR "SARS CoV" OR SARSCoV* OR Coronavirus OR Pfizer OR comirnaty OR spikevax OR moderna OR "mRNA 1273" OR "Ad26.COV*" OR Janssen OR Johnson OR 78436735* OR BNT162*) AND (vaccin*))	28,036

Table S4: Search strategy in Wiley/Cochrane Library.

Search	Query	Results
#4	#1 AND (#2 OR #3)	110
#3	(Comorbidit* OR “Co NEXT morbidit*” OR “underlying NEXT dis*” OR “co existing NEXT dis*” OR “co occurring NEXT dis*” OR “concurrent NEXT dis*” OR “underlying NEXT condition*” OR “co-existing NEXT condition*” OR “co-occurring NEXT condition*” OR “concurrent NEXT condition*” OR Multimorbidit* OR “Multi NEXT morbidit*”):ti,ab,kw	22,363
#2	(Prediabete* OR diabetes OR NIDDM OR IDDM OR T1DM OR T2DM OR diabetic* OR insulin* OR metformin* OR rosiglitazon* OR troglitazon* OR pioglitazon* OR hypoglycem* OR hypoglycaem*):ti,ab,kw	131,680
#1	((Covid* OR corona* OR ncov OR “SARS NEXT CoV” OR SARSCoV* OR Coronavirus OR Pfizer OR comirnaty OR spikevax OR moderna OR “mRNA NEXT 1273” OR “Ad26.COV*” OR Janssen OR Johnson OR 78436735* OR BNT162*) AND (vaccin*)):ti,ab,kw	1,402

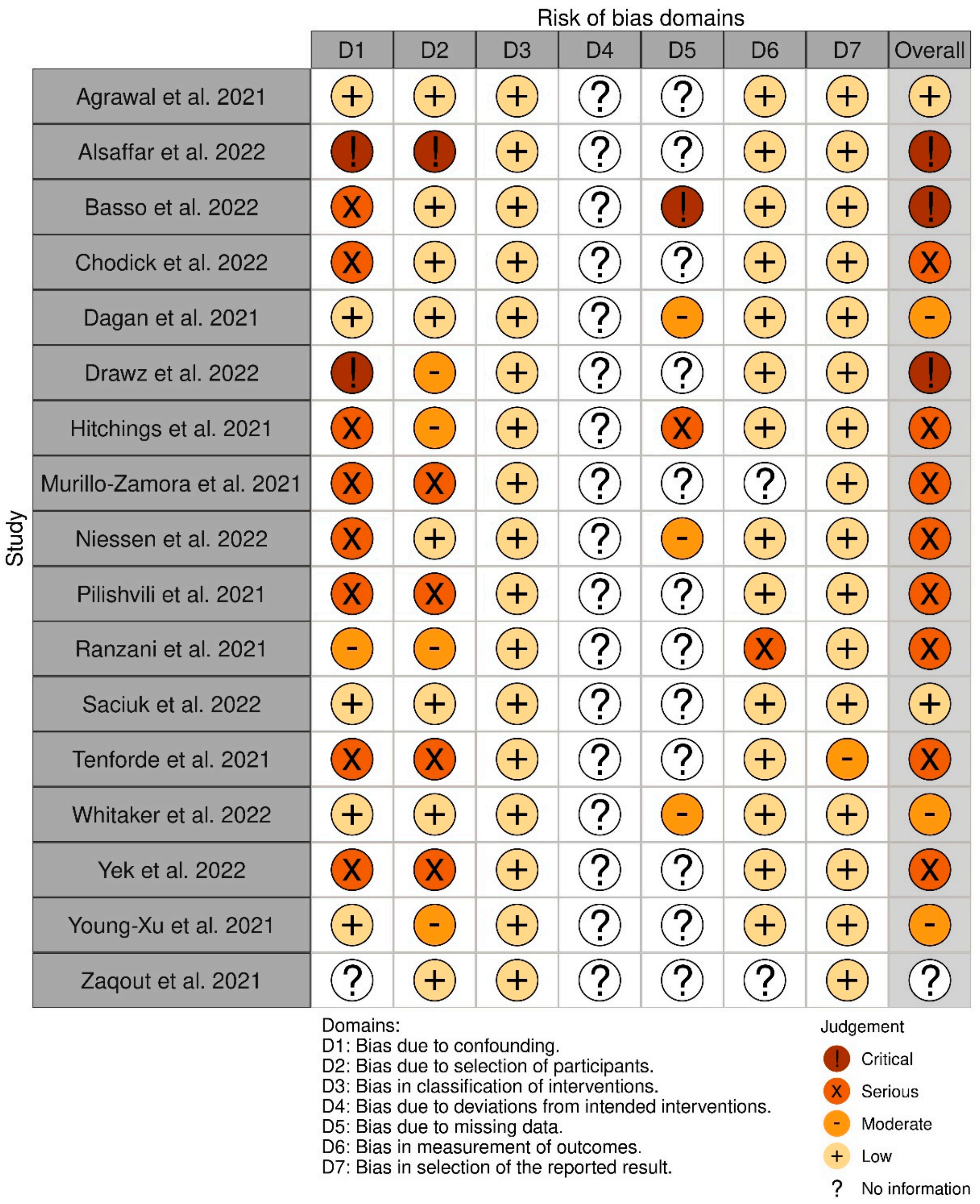

Figure S1. Risk of bias by domain and overall, for each study separately.

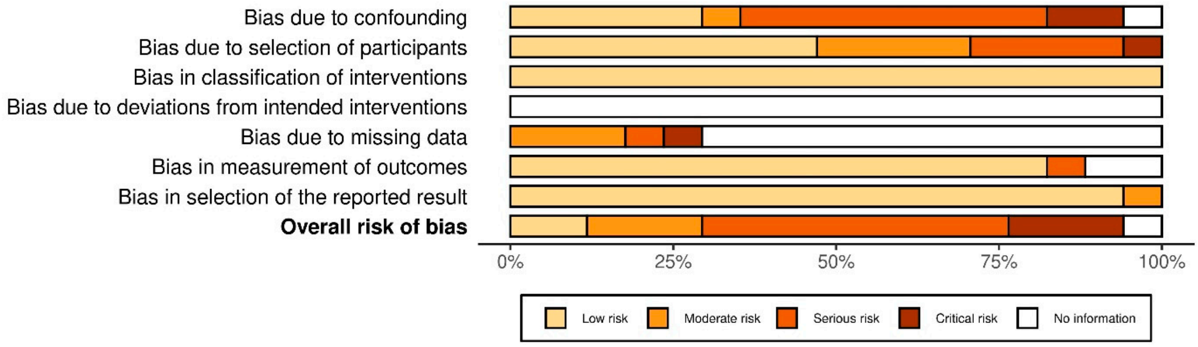

Figure S2. Risk of bias in included studies, by domain and overall.
